# Multimodal Image Integration for Epilepsy Presurgical Evaluation: A Clinical Workflow

**DOI:** 10.3389/fneur.2021.709400

**Published:** 2021-08-04

**Authors:** Liri Jin, Joon Yul Choi, Juan Bulacio, Andreas V. Alexopoulos, Richard C. Burgess, Hiroatsu Murakami, William Bingaman, Imad Najm, Zhong Irene Wang

**Affiliations:** ^1^Department of Neurology, Peking Union Medical College Hospital, Beijing, China; ^2^Epilepsy Center, Cleveland Clinic, Cleveland, OH, United States; ^3^Department of Neurosurgery, Cleveland Clinic, Cleveland, OH, United States

**Keywords:** multimodal integration, presurgical evaluation, stereoelectroencephalography, intracranial EEG, epilepsy surgery, MRI, SPECT & PET imaging, magnetoencephalography

## Abstract

Multimodal image integration (MMII) is a promising tool to help delineate the epileptogenic zone (EZ) in patients with medically intractable focal epilepsies undergoing presurgical evaluation. We report here the detailed methodology of MMII and an overview of the utility of MMII at the Cleveland Clinic Epilepsy Center from 2014 to 2018, exemplified by illustrative cases. The image integration was performed using the Curry platform (Compumedics Neuroscan^™^, Charlotte, NC, USA), including all available diagnostic modalities such as Magnetic resonance imaging (MRI), Positron Emission Tomography (PET), single-photon emission computed tomography (SPECT) and Magnetoencephalography (MEG), with additional capability of trajectory planning for intracranial EEG (ICEEG), particularly stereo-EEG (SEEG), as well as surgical resection planning. In the 5-year time span, 467 patients underwent MMII; of them, 98 patients (21%) had a history of prior neurosurgery and recurring seizures. Of the 467 patients, 425 patients underwent ICEEG implantation with further CT co-registration to identify the electrode locations. A total of 351 patients eventually underwent surgery after MMII, including 197 patients (56%) with non-lesional MRI and 223 patients (64%) with extra-temporal lobe epilepsy. Among 269 patients with 1-year post-operative follow up, 134 patients (50%) had remained completely seizure-free. The most common histopathological finding is focal cortical dysplasia. Our study illustrates the usefulness of MMII to enhance SEEG electrode trajectory planning, assist non-invasive/invasive data interpretation, plan resection strategy, and re-evaluate surgical failures. Information presented by MMII is essential to the understanding of the anatomo-functional-electro-clinical correlations in individual cases, which leads to the ultimate success of presurgical evaluation of patients with medically intractable focal epilepsies.

## Introduction

For patients with medically intractable focal epilepsies, surgery is currently their best option for seizure control (1). A successful surgical strategy strongly depends on the accurate delineation and removal of the epileptogenic zone (EZ) without unacceptable postoperative deficits. Currently, no single diagnostic test can achieve direct delineation of the EZ. The presurgical evaluation has gone towards a multidisciplinary approach, with many commonly used modalities including scalp video-EEG, Magnetic Resonance Imaging (MRI) Positron Emission Tomography (PET), single-photon emission computed tomography (SPECT), Magnetoencephalography (MEG) and functional MRI (fMRI). Intracranial EEG (ICEEG) is often necessary for further localization of the seizure onset zone when non-invasive tests alone cannot produce a conclusive localization (2). All the data from the different diagnostic tests should be interpreted in an integrated fashion, in order to generate an optimized plan for surgical resection/ablation.

Multimodal image integration (MMII) refers to the procedure that co-registers imaging data from multiple sources into the same space by an automated, computerized process. It is important to note that although results from each test can be reviewed separately within its own platform without MMII, and an experienced user can mentally fuse the localization results, MMII offers a more straightforward way for visualization, which often makes data review more convenient, more efficient and less prone to human errors. This process is especially helpful when the patient's head is positioned differently for different tests, and when anatomical landmarks are difficult to identify due to differing imaging planes. The potential value of MMII has been illustrated in a few previous studies; however, the software used were typically made in-house or based on research tools, limiting their use to academic centers (3–5). Here, we report a methodology of MMII based on an FDA-approved software platform, and detail a workflow that can be used for the clinical practice of epilepsy presurgical evaluation. We also aim to review the Cleveland Clinic experience on utilizing MMII from 2014 to 2018, with clinical values of the reported workflow exemplified by illustrative cases.

## Materials and Methods

### Patient Selection

This is a single-center retrospective study approved by the Cleveland Clinic institutional review board. We reviewed all the data reconstructed with the MMII workflow between 2014 and 2018. All patients had medically intractable focal epilepsy and were considered for ICEEG implantation and/or surgical resection. The strategy for ICEEG implantation or direct surgery was discussed in a patient management conference (PMC) based on multimodal data including scalp video-EEG, MRI, PET, ictal SPECT and MEG. If ICEEG was deemed necessary and pursued, the ICCEG findings were discussed at another PMC, in which the final surgical resection/ablation strategy was determined.

### Integration of Non-invasive Modalities

The MMII workflow was carried out on FDA-approved software platform Curry 7 (Compumedics Neuroscan^™^, Charlotte, NC, USA). The data structure was organized as databases, with each modality stored as one data folder/file within the database. DICOM images with different scanners were in general compatible with the platform, making it the most universal image format for communication among different vendors/modalities. When each modality was imported for the first time, an initialization process was needed to set the parameters, followed by image coregistration. The parameters and procedures optimized for each modality are detailed in the following sections. The workflow runs on a regular PC; the 3D rendering of cortical surface runs more smoothly with advanced graphics card.

#### Structural MRI

**Figure 1:**
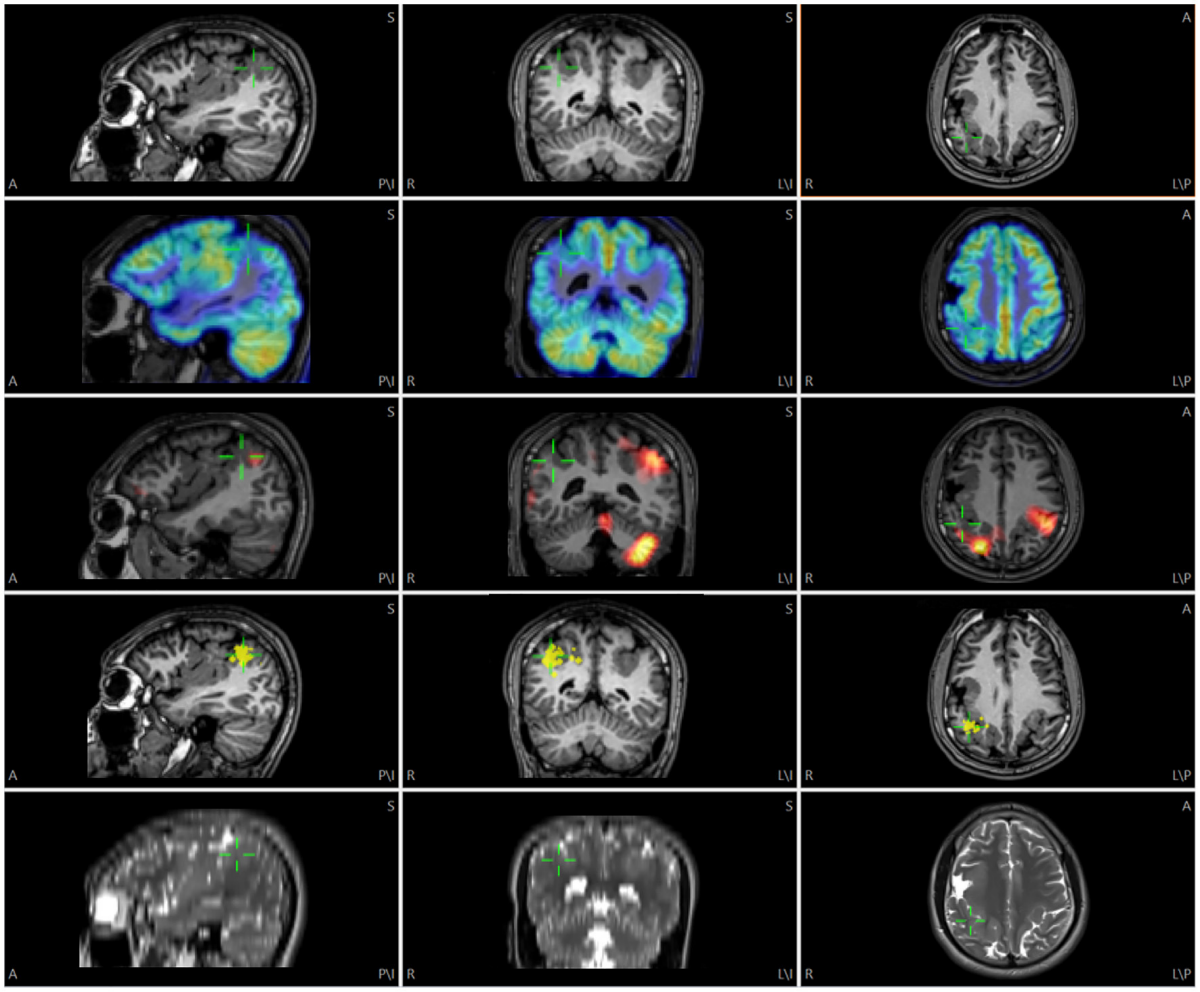
Illustration of multimodality image integration (MMII) from a patient with bilateral polymicrogyria (PMG) undergoing non-invasive presurgical evaluation. Rows from top to down: 3D T1-weighted MRI, FDG-PET, SPECT SISCOM (z score=1.5), MEG (dipole locations) and 2D axial T2-weighted TSE images. The green cursor is centered on the posterior part of the right-sided PMG that showed a tight MEG cluster, and the corresponding location is shown on all the other modalities by green cursors. For this and the following Figures, A, anterior; P, posterior; S, superior; I, inferior; L, left; R, right.

All clinical MRI scans were acquired from a 3T Siemens Skyra scanner (Siemens, Erlangen, Germany). The 3D T1-weighed Magnetization Prepared Rapid Acquisition with Gradient Echo (MPRAGE) volumetric sequence was used as the “base image volume,” which is used as an underlay, on which images from all the other modalities are coregistered to. For example, other sequences of different MRI contrasts such as 2D/3D FLAIR and 2D T2-weighted images can be coregistered to the 3D T1-weighted base image by automated full-volume registration (maximization of mutual information). This is shown in Figure 1. Similarly, post-operative MRI scans can be coregistered to compare the resection cavity with all the other modalities. Detailed structural MRI parameters were published previously (6).

#### Functional MRI

The standard clinical functional MRI (fMRI) examination consisted of four paradigms: one motor and three language tasks (covert word generation, rhyming and passive listening). For fMRI maps, motion of images obtained by an echo-planar imaging (EPI) sequence was firstly corrected using Siemens PACE software (VB15). Then, statistical maps were generated and superimposed on top of T1-weighted MPRAGE images that were typically acquired in the same fMRI scan. This T1-weighted MPRAGE image set was then imported and coregistered with the base image volume, and the same transformation matrix was applied to the statistical maps to ensure accurate coregistration.

#### PET

Fluorodeoxyglucose (FDG)-PET (FDG-PET) scans were performed on Biograph PET CT (Siemens AG, Munich, Germany). The attenuation corrected PET images were superimposed onto the base image volume by the “show thresholded” function (MRI transparency set to 75%) after adjusting PET histogram to full range. This is shown in Figures 1, 3.

#### SPECT

SPECT images were acquired on a Siemens (Erlangen, Germany) Symbia dual-head camera. The interictal perfusion image, ictal perfusion image, and a T1-weighted MPRAGE data set were used as input to the SISCOM methodology, yielding ictal-interictal subtracted z-score images that were coregistered with the T1-weighted MPRAGE (7). This T1-weighted MPRAGE image set was then imported and coregistered with the base image volume, and the same transformation matrix was applied to the z-score images to ensure accurate coregistration (Figure 1). A z-score threshold of 1.5 or 2 was typically used for interpretation (8).

#### MEG

MEG data were recorded from a 306-channel whole-head MEG system (Elekta, Helsinki, Finland) and source localization analysis was performed using the vendor NeuroMag software (Elekta, Helsinki, Finland). Individual spike analysis was performed on data segments containing visually identified epileptiform discharges (9). The location, orientation and strength of dipole sources that best fit the measured magnetic fields were calculated typically using single equivalent current dipole model at the peak of the global field power of each interictal activity (10, 11). The final results were represented by one or several clusters of dipoles superimposed on the patient's coregistered 3D T1-weighted MPRAGE. We exported the location of the MEG dipoles by printing them as high-intensity points on top of the MRI (dipole head 60%, MRI slices 5 mm, separation at print output 1 mm). The MRI DICOM images after the printing process were then imported and coregistered to the base image volume (Figure 1). The high-intensity points representing the dipole locations were then segmented and their 3D coordinates were saved as a list of localize points, and visualized in conjunction with all the other data.

#### Vasculatures

To visualize vasculatures, CT-angiography (CTA) and/or MR-angiography (MRA) images were obtained and coregistered to the base image volume; image contrast was adjusted so that when overlaid, major vessels were visible.

#### Cortical Surface

The cortical surface was generated by Freesurfer (http://www.nmr.mgh.harvard.edu/) and then imported for visualization. The pial surface files were first transformed to Curry surface format, and then spatially coregistered with the base image volume. Once the coregistration is complete, each point on the cortical surface was linked to the coronal, axial and sagittal views.

### Talairach Coordinate Definition

**Figure 2:**
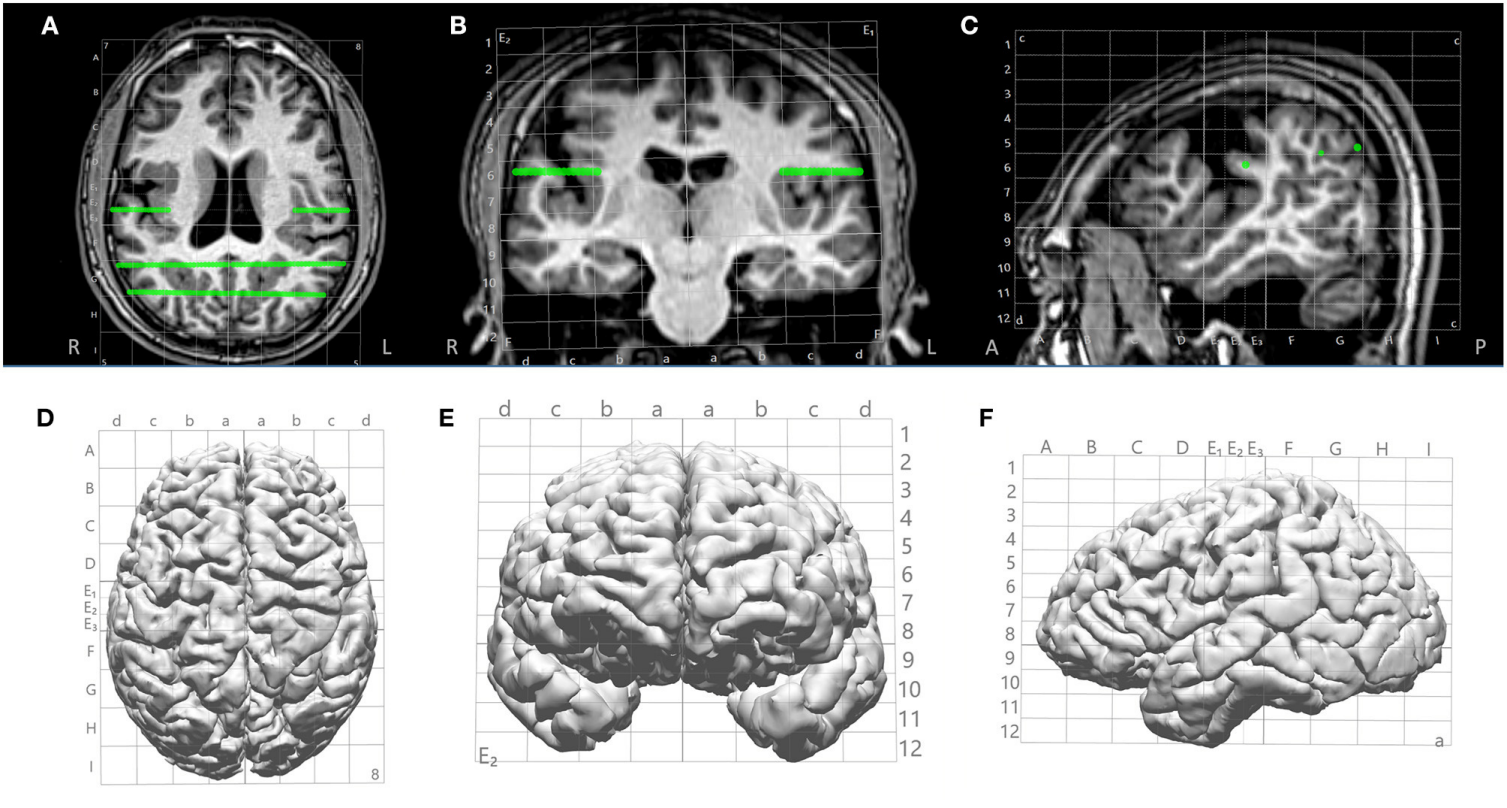
Illustration of Talairach grid defined based on AC, PC and MS points identified on the 3D T1-weighted base image. **(A–C)** 2D overlay of the Talairach grid on coronal, axial and sagittal MRI; **(D–F)** 3D overlay of the Talairach grid on cortical surface (axial, coronal and saggital). Green lines indicate electrode trajectories.

The Talairach grid was defined based on anterior commissure (AC) and posterior commissure (PC). The AC, PC and midsagittal (MS) points were first identified on the T1-weighed base image volume. Then, the boundary box of the brain was delineated on the sagittal view (anterior, posterior, superior and inferior boundaries) and axial view (lateral boundaries). The Talairach coordinates were defined with a proportional system consistent with its original definition (12). Specifically, on a sagittal view, sectors (columns) were defined as follows: the vertical AC (VCA) line and the anterior boundary of the brain was divided evenly into 4 parts: A, B, C, D. The vertical PC (VCP) line and the posterior boundary of the brain was divided evenly into 4 parts: F, G, H, I. On the sagittal view, levels (rows) were defined as follows: the distance between the AC-PC line and the superior boundary of the brain was divided evenly into 8 parts. The distance between the AC-PC line and the inferior boundary of the brain was divided evenly into 4 parts. On the coronal view, the columns were defined by dividing into 4 parts from the mid-sagittal to the most lateral boundary of the brain, for each hemisphere. Columns are named a, b, c and d from mesial to lateral. The sectors and levels were automatically shown after the initialization steps are done to identify AC, PC, MS, VCA, VCP and brain boundary box. This is illustrated in Figure 2.

### SEEG Trajectory Planning

**Figure 3:**
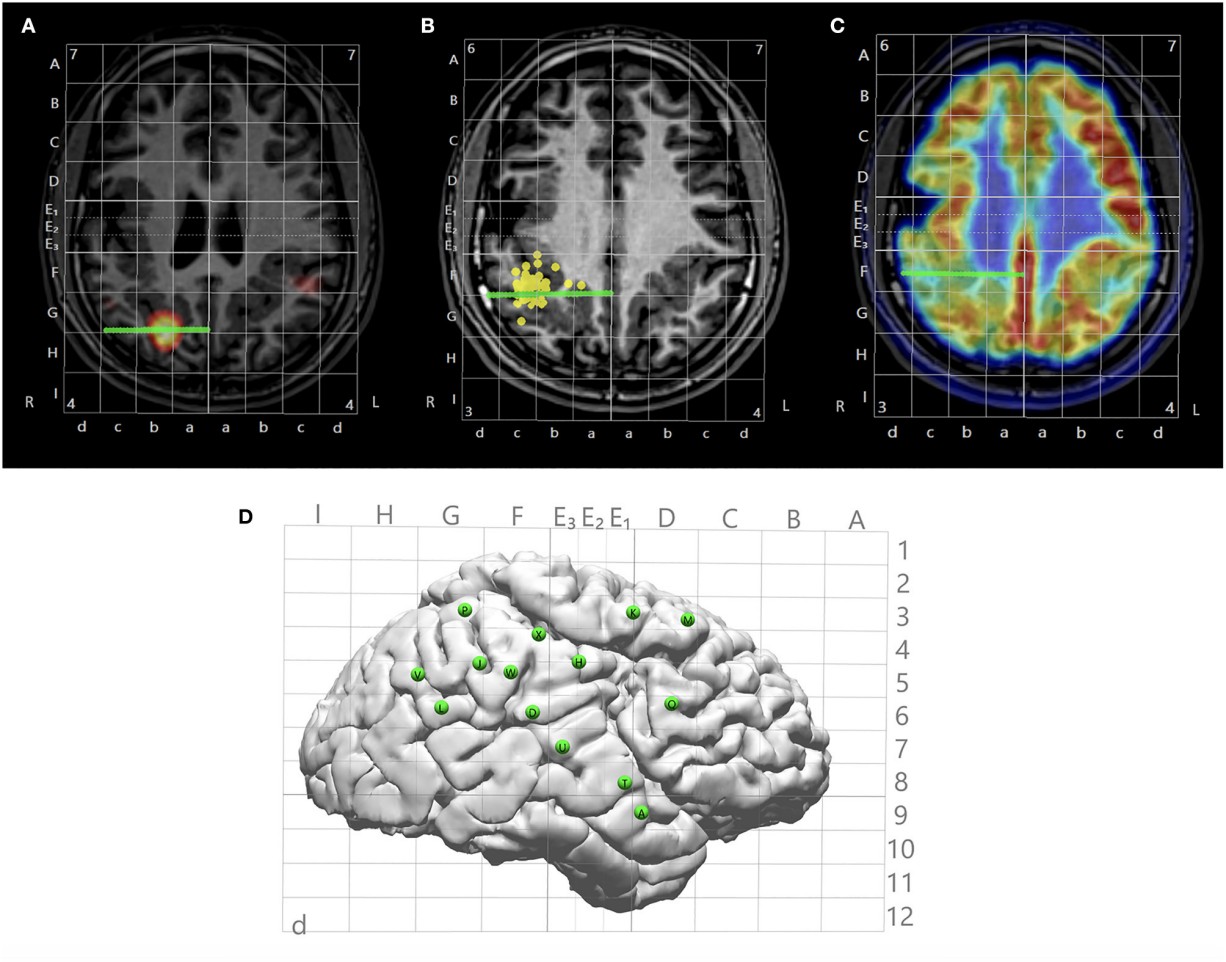
Illustration of multimodal integration assisting trajectory planning. **(A)** A trajectory planned to target a hyperperfusion region shown by subtraction ictal SPECT co-registered with MRI (SISCOM) analysis. **(B)** A trajectory planned to target a tight MEG cluster. **(C)** A trajectory planned to target hypometabolic regions on the PET. **(D)** Entry points of all planned trajectories shown on the cortical surface with Talairach grid overlaid.

After all images from the non-invasive modalities were fused and Talairach coordinates defined, trajectories of SEEG could be planned based on any chosen modality that had localization value to the case (Figures 3A–C). An entry point and a target point were defined for each electrode, and the trajectories were created as a straight line connecting these two points (as shown in Figures 2, 3), avoiding major vasculatures (shown by the CTA or MRA) especially at the entry point. The Talairach grid can be used as a reference for the definition of the entry and target points. All planned trajectories can then be displayed on the cortical surface to examine their coverage (Figure 3D), and can be exported as high-intensity lines on the T1-weighed base image volume as DICOM images, which can be further incorporated into the neuronavigation systems, e.g., ROSA (Medtech, Montpellier, France) and Brainlab (Brainlab, Feldkirchen, Germany), to facilitate implantation in the OR.

### Reconstruction of ICEEG Implantation

**Figure 4:**
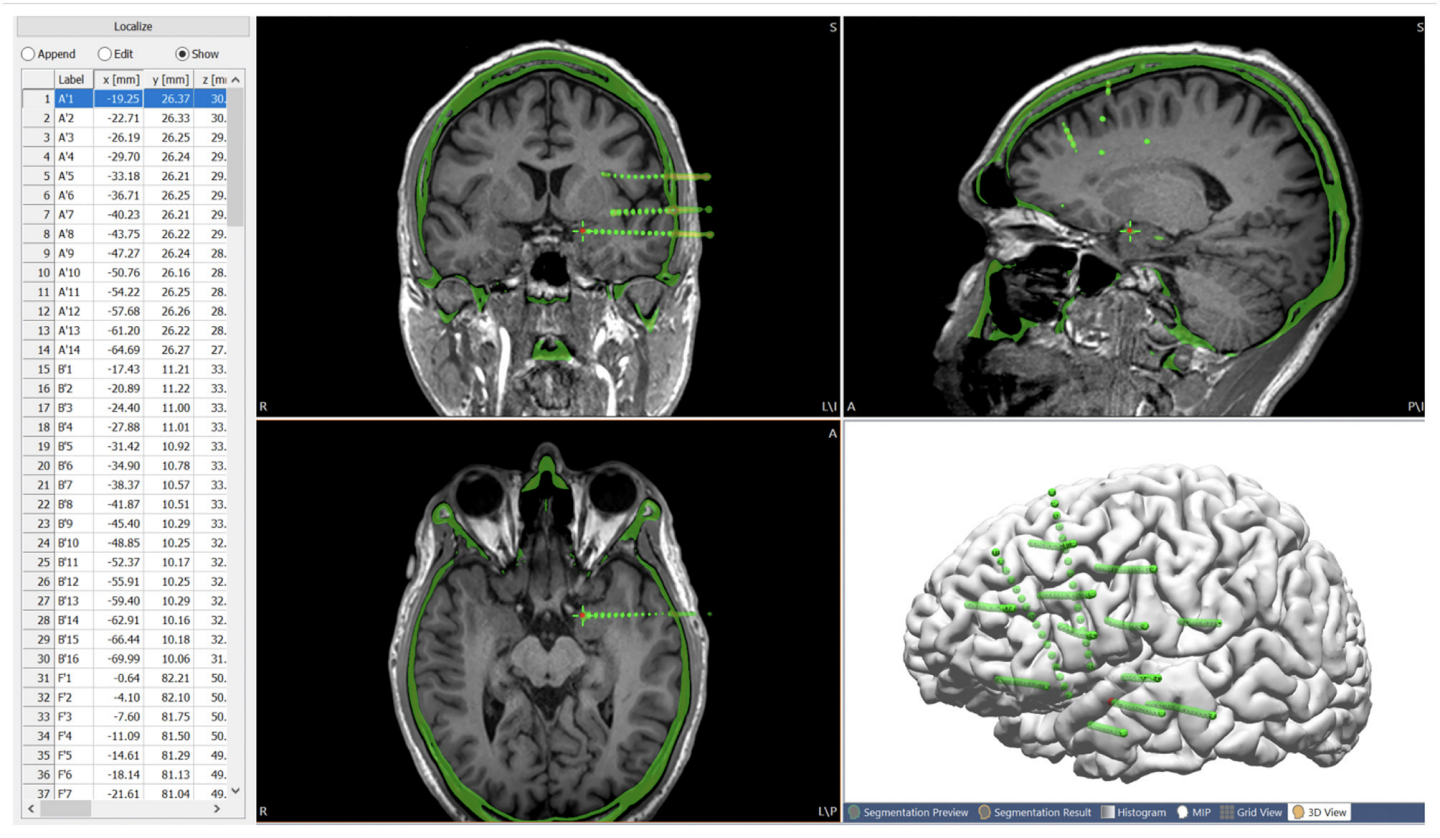
Post-SEEG-implantation CT images co-registered with the T1-weighted base image, with all the electrode contacts segmented from the CT (center of highest intensity) and stored as a list of localize points with coordinates for the electrode contacts. Green spheres indicate extracted electrode contacts. Red contact indicates the current electrode contact (highlighted on the list of localize points), the location of which is synchronized on the MRI and CT.

A high-resolution CT was taken immediately after the ICEEG implantation (SEEG or subdural grid and depth implantation), and the images were used to indicate the location of the implanted electrodes. After fusing with the T1-weighted base image, all electrode contacts were segmented from the CT (the center of maximal intensity was taken as the electrode contact location), and stored as a list of localize points with coordinates for the electrode contacts. The contacts were individually named and displayed in an interactive fashion. This is shown in Figure 4.

### Analysis of ICEEG Data in Conjunction With Non-invasive Modalities

Since the non-invasive modalities were all registered to the same space of the T1-weighted base image volume, once the post-implantation CT image was co-registered to the base image volume, direct comparison between the ICEEG data and the non-invasive evaluation data was immediately feasible. This facilitates straightforward comparisons of the localization results from non-invasive modalities with the interictal and ictal findings from ICEEG.

### Planning Resective/Ablative Surgery

**Figure 5:**
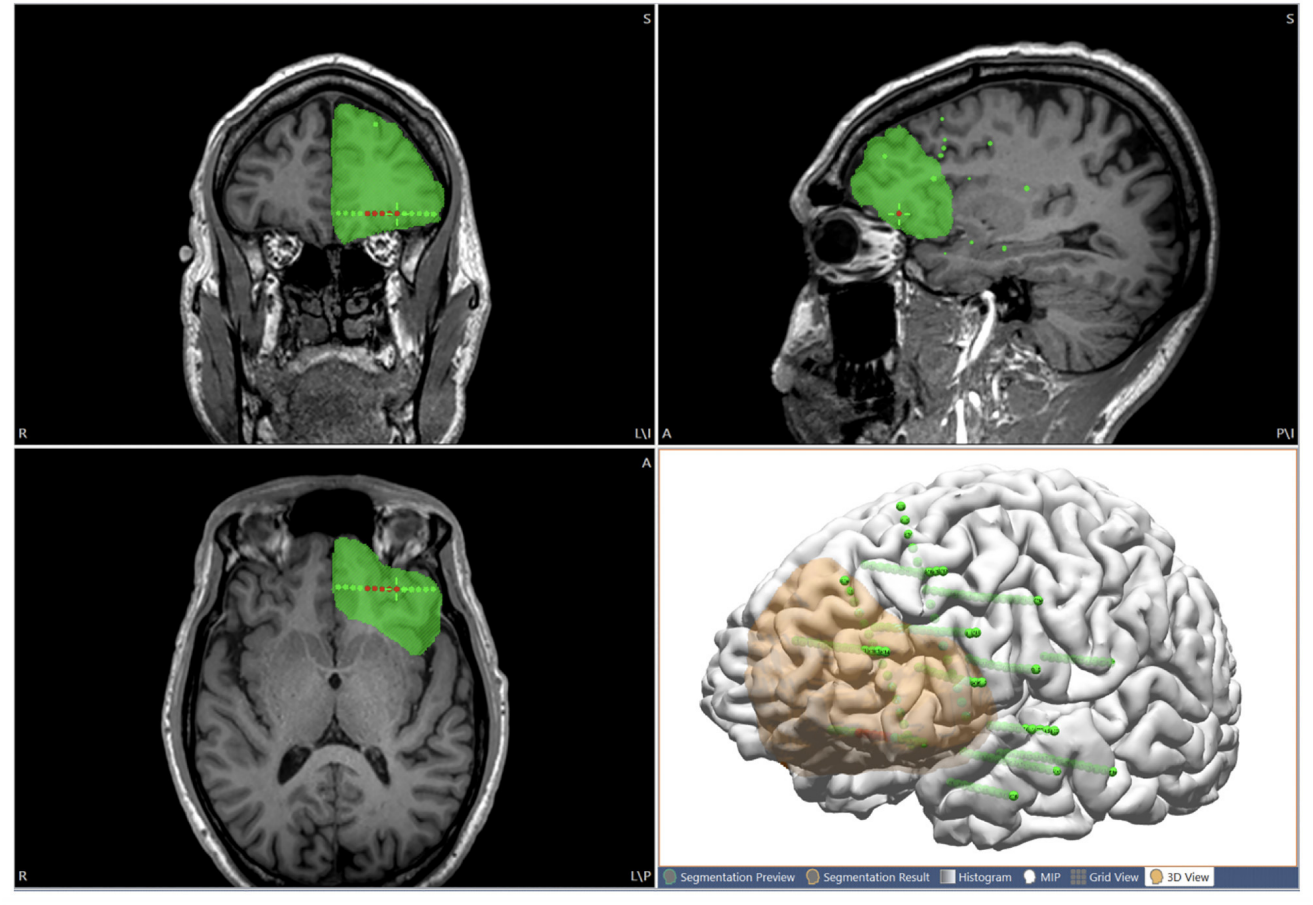
Illustration of using the multimodal image integration workflow for planning resection. The proposed resection area is interactively drawn on the MRI (coronal, axial and sagittal views), and can also be shown in 3D view together with the cortical surface (orange region). Green spheres indicate all the implanted SEEG electrodes. Red spheres indicate seizure-onset contacts.

After considering all the non-invasive and ICEEG data, the proposed resection area can be interactively drawn (as “pass markers”) on the 3D T1-weighted base MRI (on 2D coronal, axial and sagittal views) to include ictal onset contacts and other pertinent areas thought to be the putative epileptogenic zone. The drawings can also be segmented as voxel mesh shown in 3D view together with the cortical surface (Figure 5). Finally, the proposed resection area can be exported as a high intensity area printed on the 3D T1-weighted base image volume, and exported as DICOM images to be taken by the neuronavigation system to guide resection/ablation.

### Evaluating Reoperation

For patients with recurring seizures after the first operation, re-evaluation for a potential reoperation can be a worthwhile attempt (13, 14). To this end, the postoperative MRI can be coregistered with the preoperative T1-weighted base image volume, to assess the spatial relationship between the resection cavity and all available data from the prior and current evaluations.

### Validating MRI Postprocessing Findings

MRI postprocessing using the voxel-based morphometric analysis program (MAP) method (15) generates statistical maps depicting morphological characteristics of the brain that are frequently helpful for detecting subtle lesions. The MAP process generates several output files, including the gray-white matter junction, extension and cortical thickness z-score maps, as well as the coregistered 3D T1-weighted MPRAGE images. This T1-weighted MPRAGE image set was co-registered with the base image volume, and the same transformation matrix was applied to the z-score maps.

## Results

**Figure 6:**
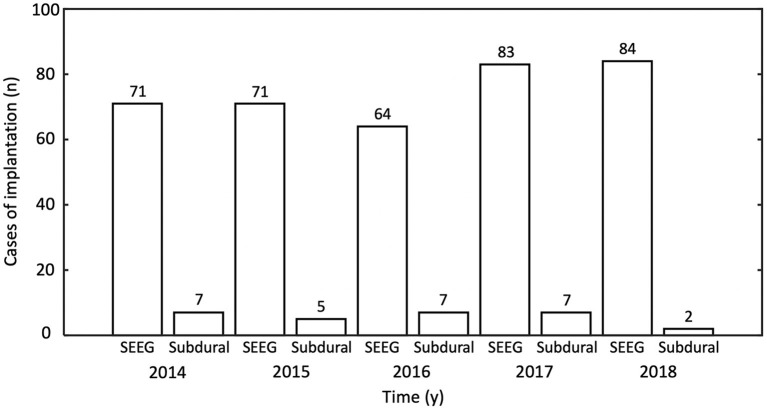
Number of intracranial EEG (ICEEG) studies after multimodal image integration was performed, tabulated by year and type of ICEEG. y, year; SEEG, stereo-EEG.

In total, 467 patients underwent MMII from 2014 to 2018. All patients who received ICEEG had MMII; in addition, MMII was also used to generate pre-implantation hypothesis for ICEEG, and in the face of highly concordant non-invasive data, can often lead to direct surgery. In the 467 patients who underwent MMII, all (100%) had MRI; 425 (91%) had PET; 334 had SPECT (72%) and 264 (57%) had MEG. Ninety-eight patients (21%) had prior neurosurgery, including resective/disconnective/ablative surgery for epilepsy, other resections for brain tumor/vascular malformation/post-traumatic lesions, etc. After MMII was performed, 425 of the 467 patients (91%) further underwent ICEEG implantation, including 396 cases with SEEG, 28 cases with subdural grids and depth electrodes placement and 1 case with subdural and depth electrodes implantation followed by SEEG (numbers per year are detailed in Figure 6). Among the 425 patients who had ICEEG, 256 patients (60%) had negative MRI, and 283 patients (67%) had extra-temporal lobe epilepsy (ETLE). For the 42 patients who underwent MMII but did not undergo ICEEG, 37 of them underwent surgery directly due to the concordance of non-invasive findings obliviating the need for ICEEG; 5 declined ICEEG or lost follow-up.

A total of 351 cases underwent further surgery after MMII (male-to-female ratio = 1.2:1; mean age = 29.4 years, range = 5–69; mean seizure history = 15.7 years, range = 1–55; negative MRI in 56%; ETLE in 64%). Among them 285 patients had resections, 26 patients had neuromodulation and 40 patients had laser ablation. Among the 269 patients with at least one-year postoperative follow-up, 134 patients (50%) had remained completely seizure-free at 1 year. Pathology from the resected 285 patients included FCD in 168, hippocampal sclerosis in six, tumor in six, vascular malformation in two, gliosis in 46, double pathology in 21 and other non-specific findings in 36.

### Illustrative Case 1

**Figure 7:**
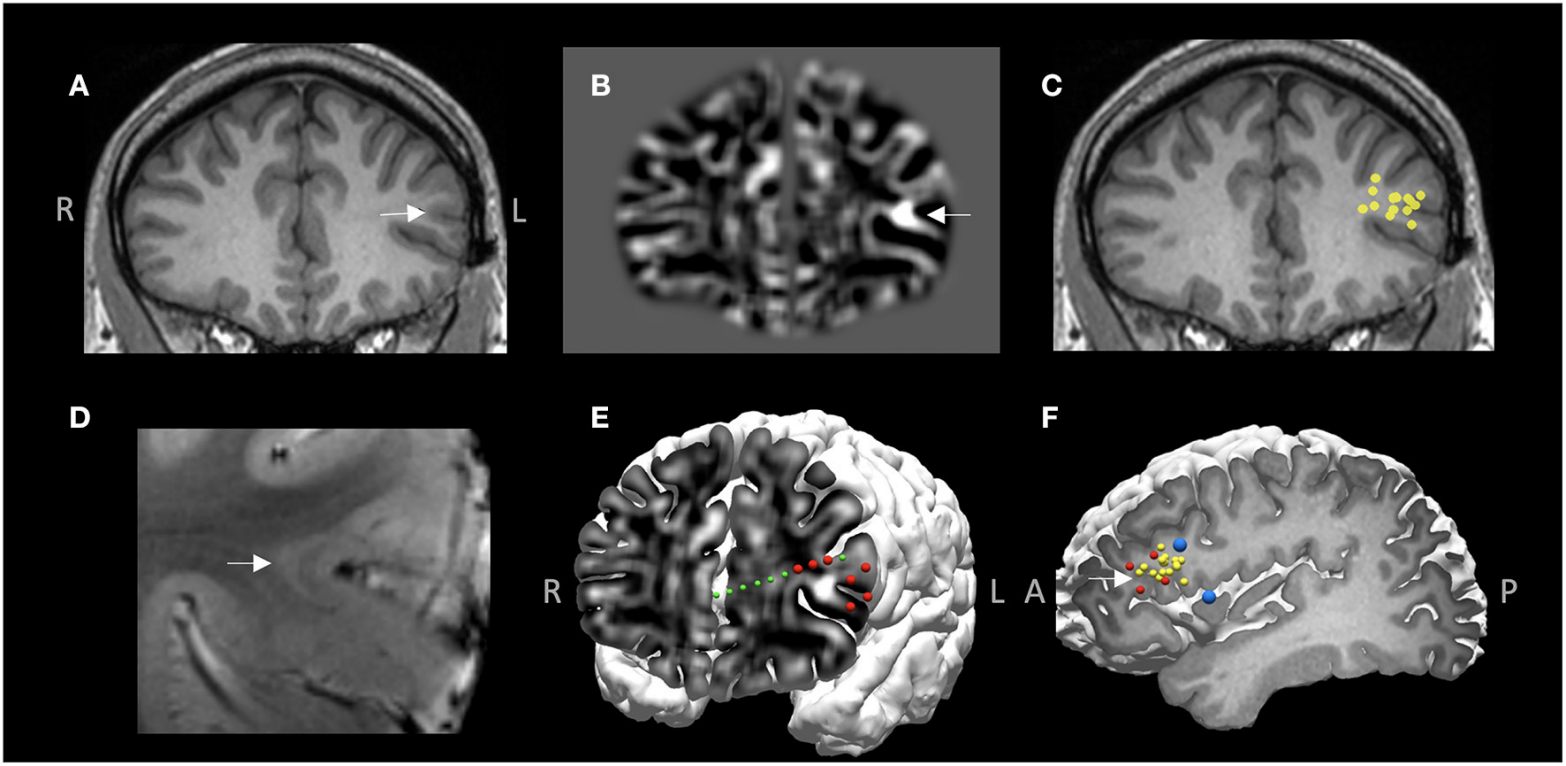
MMII findings in Case 1. **(A)** 3T T1-weighted MRI on coronal plane. **(B)** Z-score map of gray-white matter junction from MAP processing based on the 3T T1-weighted images, shown on the same coronal plane as **(A)**, demonstrating a subtle structural abnormality with gray-white blurring, likely focal cortical dysplasia (FCD). **(C)** A tight MEG dipole cluster shown on the same coronal plane as **(A,B)**, illustrating the MEG cluster overlapped well with the subtle structural abnormality. **(D)** 7T T2*-weighted GRE image corroborating the finding of a subtle FCD lesion with transmantle sign. **(E)** 3D view of ictal onset ICEEG electrode contacts (red spheres), both on the depth electrode (green spheres) and the subdural grid (for best visualization, only the onset electrode contacts are shown, not all the implanted grids), “sandwiching” the subtle structural abnormality. z-score map of gray-white matter junction from MAP processing is fused with the cortical surface to reveal the 2D-3D spatial relationship of the subtle lesion and the overlaying cortex as well as the ICEEG findings. **(F)** The tight MEG cluster (yellow spheres), ictal onset (red spheres) and language regions shown on cortical stimulation (blue spheres). White arrow indicates the location of the subtle structural abnormality in **(A,B,D,F)**. This patient underwent a focused left frontal lesionectomy, had no speech deficits postoperatively and remained seizure-free for 4 years at the most recent follow up. Histopathology showed FCD type IIb.

A 26-year-old right-handed male with medically intractable focal epilepsy since the age of 4 was evaluated for surgery. Seizure semiology was characterized by axial tonic → left face clonic → right arm clonic → secondary generalization. Both interictal and ictal scalp EEG localized to the left frontotemporal region. The 3T MRI was negative. MRI postprocessing using the MAP method on both 3T clinical MRI and 7T research MRI showed a suspicious lesion with subtle gray-white blurring at the bottom of an accessory sulcus in the left pars triangularis (Figure 7B), which was then confirmed by re-review of the original MRI scans (Figures 7A,D). Among all the other non-invasive modalities, interictal MEG was the most localizing, showing a tight cluster in the left inferior frontal region (Figure 7C), overlapping with the subtle structural lesion. At the multidisciplinary PMC, ictal onset arising from the left inferior frontal and opercular regions was hypothesized. ICEEG was thought necessary to confirm this hypothesis and precisely delineate resection extent, especially for concerns of potential language deficit. ICEEG confirmed the ictal onset in the area closely adjacent to the subtle lesion in the left inferior frontal gyrus (Figure 7E). The relative location among the structural lesion, MEG cluster, ICEEG ictal findings and cortical stimulation results for language are demonstrated in Figure 7F. This patient underwent a focused left frontal lesionectomy, after further being guided by both electrocorticography and functional mapping intraoperatively. He had no speech deficits postoperatively and remained seizure-free for 4 years at the most recent follow up. Histopathology showed FCD type IIb.

### Illustrative Case 2

**Figure 8:**
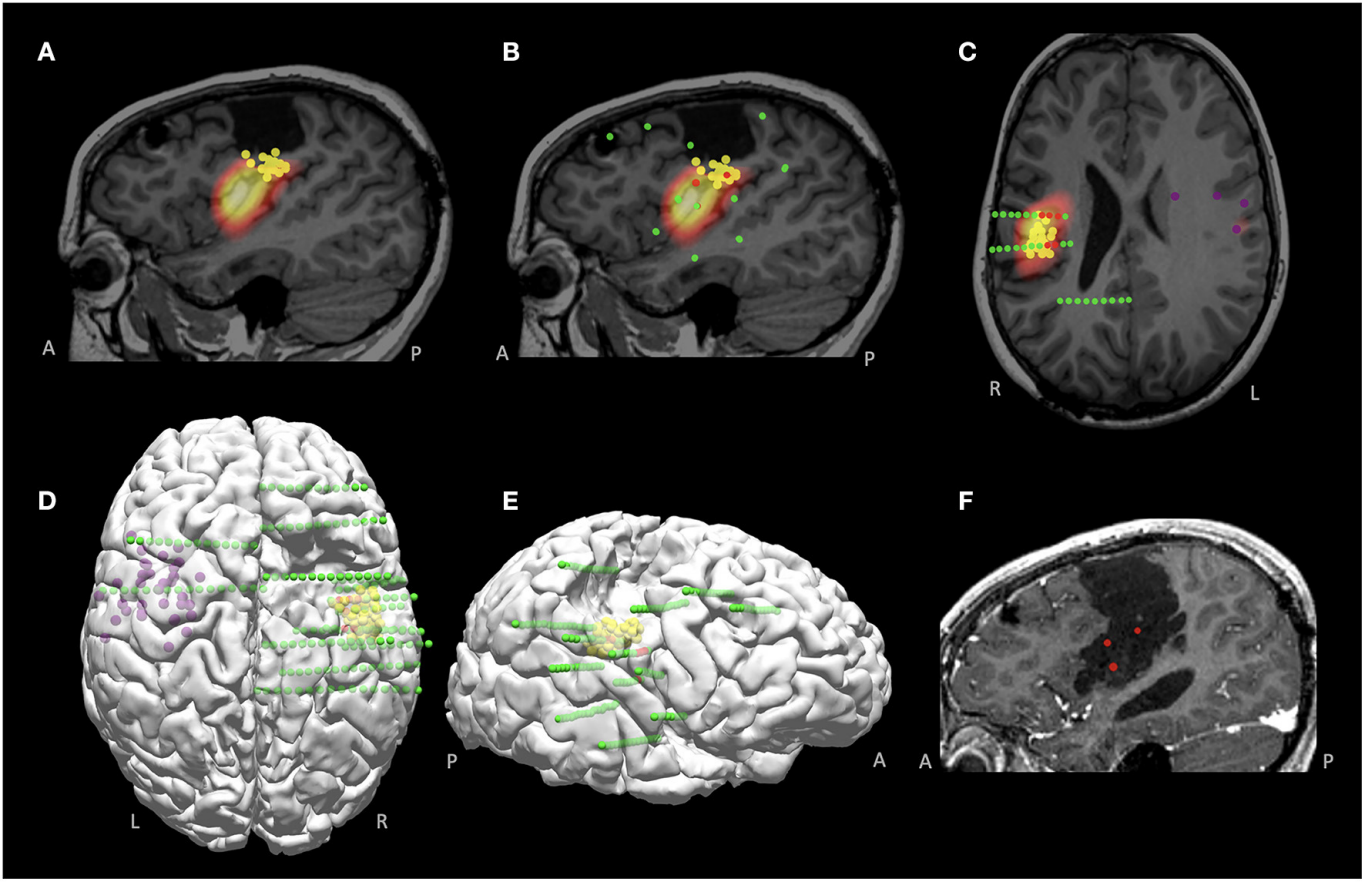
MMII findings in Case 2 with a previously failed epilepsy surgery. **(A)** Co-localized MEG and SPECT findings, around the inferior margin of the previous resection, shown on the sagittal plane. **(B,C)** Sagittal and axial views of the SEEG implantation, overlaid with the MEG findings (yellow spheres indicate right-sided tight MEG luster, purple spheres indicate left-sided loose MEG cluster). Green spheres indicate all implanted electrodes and red spheres indicate ictal onset, which was highly concordant with the co-localized MEG and SPECT region. **(D,E)** 3D views (top view and right-sided view) of the findings from MEG and SEEG, visualizing the spatial relationship with the prior resection. **(F)** Post-operative MRI after the re-operation, showing extension of the prior resection, including the SEEG ictal onset and the co-localized MEG and SPECT region. The patient became seizure-free at the most recent 1-year follow up, without new neurological deficits. Histopathology showed remote ischemic damage.

A 11-year-old right-handed female presented with a history of medically intractable seizures starting at the age of 4 years. Birth and developmental history were unremarkable. She had previously undergone right frontal and central resection at an outside hospital at the age of 9 years without seizure reduction and developed left hemiparesis postoperatively. The majority of seizures started during sleep and semiology showed bilateral tonic stiffening, followed by face clonic and then generalized clonic seizures. Scalp EEG showed interictal sharp waves involving the right parietal-temporal regions, and ictal pattern arising from the right posterior temporal-parietal region with rapid spread bilaterally. No focal abnormality was found on the previous and repeated MRIs. Interictal MEG activity was localized to the right frontoparietal operculum, on the inferior margin of the prior resection cavity (Figure 8A); another loose cluster was shown on the left precentral region extending rostrally to the premotor area and insula. Ictal SPECT showed hyperperfusion in the right posterior insular and frontoparietal operculum regions (Figure 8A). SEEG implantation was predominately right-sided, with trajectories designed to mainly cover the areas surrounding the prior resection, especially the areas co-localized by MEG and SPECT (Figures 8B,C); two electrodes were implanted on the left side driven by the loose MEG cluster on the left (Figure 8D). SEEG onset showed a stereotyped pattern consisting of beta activity in the right posterior insula and parietal operculum regions, highly concordant with the MEG and SPECT findings (Figures 8B–E). An extended resection was performed to include the ictal onset indicated by the SEEG (Figure 8F). The patient became seizure-free at the most recent one-year follow up, without new neurological deficits. Histopathology showed remote ischemic damage.

### Illustrative Case 3

**Figure 9:**
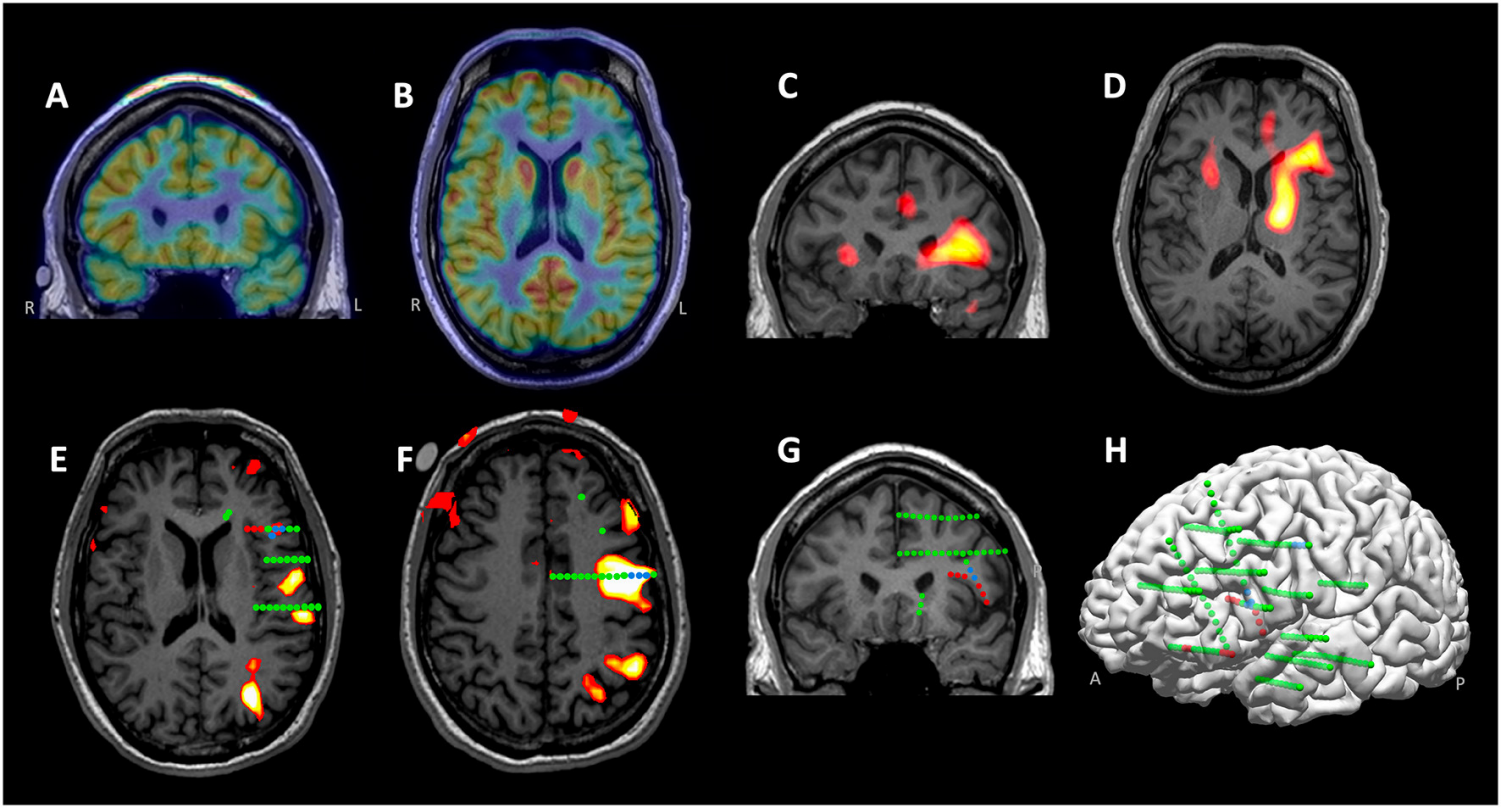
MMII findings in Case 3. **(A,B)** Prominent hypometabolism in the left inferior frontal region revealed on the coronal and axial PET. **(C,D)** Ictal SPECT (coronal and axial) showing bilateral hyperperfusion, more involved in the left inferior frontal cortex. **(E,F)** 3T language fMRI activation (during a covert word generation task) overlaid with SEEG electrodes, shown on two different MRI slices key to the cortical stimulation findings. SEEG Ictal onset arose from the left frontal operculum with network involvement of the orbitofrontal cortex, as denoted by red spheres. Speech arrest was elicited by stimulation at high intensity in the white matter tract underlying pars triangularis and in the dorsal aspect of pars opercularis (both shown with blue spheres). **(G,H)** Left-sided SEEG implantation shown on 2D coronal slice and 3D view. The implantation (all implanted electrodes shown with green spheres) covered the left frontal, temporal, insular and opercular regions, especially with key trajectories targeting the PET and ictal SPECT localizations. Following intraoperative language mapping, the patient underwent a resection of the left orbitofrontal cortex, the anterior short gyrus of the insular and frontal operculum. He remained seizure free for 18 months at the most recent follow up, and had no language deficits since surgery. Histopathology showed FCD type IIb.

A 30-year-old, right-handed male presented with medically intractable epilepsy for 21 years, with daily nocturnal seizures characterized by tremor-like twitching of the right arm, occasionally followed by asymmetric facial pulling more on the right, and then dialeptic seizures. Epileptiform activities were seen over the left frontotemporal region on scalp EEG interictally and ictally. Both 3T/7T MRI and MAP analysis were normal. PET revealed prominent hypometabolism in the left inferior frontal region (Figures 9A,B). Ictal SPECT showed bilateral hyperperfusion but more involved in the left inferior frontal cortex (Figures 9C,D). MEG was negative. fMRI demonstrated left hemisphere representation for both receptive and expressive language (Figures 9E,F show activation from the covert word generation task). Based on the non-invasive findings, the patient underwent left-sided SEEG implantation, aiming to explore the left frontal, temporal, insular and opercular regions, especially with key trajectories targeting the PET hypometabolism and ictal SPECT hyperperfusion regions (Figures 9E,F). Ictal onset arising from the left frontal operculum with network involvement of the orbitofrontal cortex were identified on the SEEG (Figures 9G,H, red spheres). Speech arrest was not elicited on the ictal onset contacts in the left frontal operculum, but instead, was elicited by stimulation at high intensity (7 mA) in the white matter tract underlying pars triangularis (as expected), and in the dorsal aspect of pars opercularis (Figures 9E–H, blue spheres); both locations had overlap with the fMRI activation areas. Therefore, language was thought to be located posterior and superior to the ictal onset contacts. Following intraoperative language mapping, the patient underwent a resection of the left orbitofrontal cortex, the anterior short gyrus of the insular and frontal operculum. He had remained seizure-free for 18 months at the most recent follow up, and had no language deficits since surgery. Histopathology showed FCD type IIb.

## Discussion

Surgical treatment of intractable focal epilepsy is challenging, especially for patients with non-lesional MRI, previously failed surgery, or deep epileptogenic foci. The process of integrating non-invasive and invasive data from all available diagnostic modalities in one single platform, is crucial to generate, inform, dispute or add to the electroclinical hypothesis before arriving at a surgical strategy. The ability to display modalities both 2D and 3D provides an optimal way to interpret the epileptic networks based on the “anatomo-electro-clinical” concept (12), which is particularly relevant for SEEG. We highlight the clinical values of MMII in the sections below.

### Guide Invasive Electrode Placement

ICEEG is frequently performed for epilepsy patients with negative MRI (63% in our cohort) or patients with ETLE (64% in our cohort), when non-invasive tests are not concordant or inconclusive for the localization of EZ (16). SEEG is more frequently performed than subdural grid and depth implantation, occupying 93% in our cohort. Due to the “tunnel vision” nature of SEEG, its success heavily depends on the preimplantation process, where facilitation from MMII is key. Each diagnostic tool has its own strengths and weaknesses, due to the different properties they measure from the epileptic network. In a prior study, we found a strong quadratic fall-off relationship between the amplitude of spikes seen on SEEG and distance of SEEG contact and MEG cluster; with a distance between SEEG and MEG cluster being 10 mm, the amplitude of SEEG spikes would drop to 40% (17). This drop-off suggests that an electrode contact slightly farther away from the source than 10 mm may be falsely negative. Thus, the invasive implantation trajectory design should accurately target the non-invasive localization results, to ensure the most thorough investigation of the epileptic network (18). In a prior study, substantial changes were seen in the overall strategy of ICEEG and the actual trajectory planning of ICEEG, after MMII data was shown (3). In the 44 patients studied, disclosure of the MMII data led to a change in surgical strategy in about 1/3, including addition and subtraction of electrodes, addition of grids, and going directly to resection. In terms of detailed trajectory planning, about 80% of cases had a change after disclosure of MMII data; most of these trajectory changes occurred in patients with SEEG and not for subdural grid implantation (3). Along the same line, the importance of accurately targeting the non-invasive localization results is illustrated here in Case 1, where the concordant findings of the subtle structural lesion and the MEG cluster contributed to a focal target for surgical resection, highlighting the role of using MRI postprocessing for subtle lesion detection and correlating with electrophysiological measures (19, 20). Case 3 illustrates a scenario where none of the structural imaging modalities, including 3T MRI, 7T MRI and postprocessing, generated positive results. However, based on the concordant functional imaging data (PET and ictal SPECT), again illustrated in 2D and 3D by MMII, a successful SEEG implantation was performed to target both the presumed EZ and potential language area, leading to seizure-free outcome with no language deficit. In the face of a completely non-lesional MRI, whether or not the patient could be a good surgical candidate would have been debatable; however, the concordant functional imaging findings led to much stronger implantation hypotheses (21). Similarly, the SEEG placement strategy in Case 2 was guided by the converging data provided by MEG and ictal SPECT, again illustrated in 2D and 3D by MMII. In both Case 1 and Case 2, the subsequent concordant SEEG ictal onset and favorable postoperative seizure outcome also support previously reported findings that MEG clusters, especially the tight clusters, should not be overlooked when planning SEEG (22).

### Assist Interpretation of Non-invasive and Invasive Data

Establishment of anatomo-electro-clinical correlations necessary for the localization of the EZ is crucial in the course of the invasive evaluation. With MMII, complex spatial relationships among the implanted electrodes, structural and functional imaging findings, and eloquent regions of interest can be more conveniently visualized. Therefore, interpretation of the interictal and ictal invasive findings could be straightforwardly carried out in conjunction with the non-invasive modalities. For example, when the coverage of invasive EEG is judged to be inadequate, the integrated MEG findings can provide a synoptic view of whole-brain activities, and “fill in the gaps” where there are no electrodes implanted (23). As another example, concordant results from one test could trigger a focused re-review of another, as illustrated in a previous study where results from MEG guided re-review of the structural MRI (24). Not infrequently, such re-review can change the MRI study from negative to positive, when subtle structural changes are brought out by concordant electrophysiological localization results. Therefore, reviewing multimodal data should be carried out in an iterative fashion, allowing the modalities to inform one another. Lastly, the site of eloquent cortex identified by electrical stimulation can also be directly overlaid, revealing a clear spatial relation between the eloquent brain regions and the ictal onset zone/propagation sites. This was clearly illustrated in Case 1 and Case 3, where the presumed EZ was in the proximity of the language area. 3D reconstruction of the ictal onset zone, the language fMRI activation maps and the cortical stimulation results provided a clear view of anatomical relationship between the hypothesized EZ and the language area, leading to the successful planning of a focal resection strategy.

### Inform Evaluation of Surgical Failures

Re-revaluation of patients with previously failed epilepsy surgery is a challenging process, and this population occupied 21% in our cohort. Re-operation might have increased risks of neurological or surgical complications, and in the meantime faced with more difficulties in localizing EZ or delineating the margins of EZ (25, 26). MMII can often help in this process by putting together all the data collected from the previous evaluation and the current evaluation, to illustrate the spatial anatomical relationship between the resection cavity and the presumed EZ in 2D and 3D. This may reveal whether the prior resection indeed included the areas intended to be resected, and whether the previous invasive evaluation had sufficient coverage. If the resection/ablation indeed completely include all the intended areas, secondary hypothesis could be explored based on the data from the previous and current evaluations. Once these factors are identified, consideration of further steps for re-operation may be more straightforward. This is well-illustrated in Case 2, where the ictal onset identified on SEEG was localized to the region just inferior to the prior resection cavity, overlapping with the area co-localized by the tight MEG cluster and SPECT hyperperfusion. These results led to a successful extension of the prior resection, which gave the patient seizure freedom without any new deficit. This finding is also consistent with a previous study that showed concordant MEG and ictal SPECT could lead to successful re-operation in patients who had a prior failed epilepsy surgery (13).

### Validate Novel Imaging Findings

The past decade has seen tremendous growth of novel imaging and image processing techniques. Before adopting these techniques into clinical use, validation of the results is crucial to minimize the false positive findings. This was illustrated in Case 1, where the subtle lesion detected by MRI postprocessing was validated by the MEG and ICEEG standard-of-care findings. Other novel structural imaging techniques, such as ultra-high-field structural 7T MRI and MR fingerprinting, can also be compared and validated by findings from clinical tests (non-invasive and invasive) within the MMII platform, to gain more insight on the epileptogenic relevance of the additional structural findings yielded by the new imaging techniques (6, 27).

### Limitations

Our study aimed at reporting the MMII methodology and illustrating the clinical value of MMII, and was not designed to quantitatively evaluate the efficacy of MMII in epilepsy surgery, due to its retrospective nature. This is an inherent limitation of the current study. While fully understanding this limitation, it may be helpful to compare the postsurgical seizure outcome in a similar cohort from literature. The best study for this comparison is perhaps the one we previously published (22), which examined a similar cohort in our center from 2008 to 2013 (all patients had SEEG), and showed seizure freedom in 44% at one-year follow-up. This study was performed right before 2014 when we consistently started to perform MMII with the workflow outlined in this study. Comparing to the seizure outcome data from the prior study, our current study shows an increased seizure-free rate of 50%. We could not, however, solely attribute this increase to MMII, as surgical outcomes are based on a number of complex factors, and the differences in patient selection in the two studies may preclude a fair comparison. Overall, how much clinical impact our methodology of MMII has on seizure outcomes, as compared to the scenario when no MMII is used, could only be measured using prospectively designed study which is very much warranted (3).

## Conclusion

Multimodal image integration is a valuable tool to understand the anatomo-functional-electro-clinical correlations in individual cases during the presurgical evaluation process. It substantially enhances the assessment of non-invasive modalities, providing essential information for generating surgical hypotheses. It also assists planning of SEEG electrode trajectories, interpretation of invasive data, design of the final resection, re-evaluation of surgical failures and validation of novel research tools. All these aspects could contribute to the ultimate success of presurgical evaluation of patients with medically intractable focal epilepsies. Multimodal image integration should be performed as a standard process and take a more prominent role in epilepsy presurgical evaluation.

## Data Availability Statement

The datasets generated for this article are not readily available because the data may contain protected health information for patient care. Requests to access the datasets should be directed to wangi2@ccf.org.

## Ethics Statement

The studies involving human participants were reviewed and approved by Cleveland Clinic IRB. Written informed consent from the patients/ participants or patients/participants legal guardian/next of kin was not required to participate in this study in accordance with the national legislation and the institutional requirements. Written informed consent was obtained from the individual(s) and/or minor(s)' legal guardian/next of kin for the publication of any potentially identifiable images or data included in this article.

## Author Contributions

LJ: data collection and analysis and manuscript drafting. JC: data analysis and manuscript drafting. JB, HM, WB, and IN: clinical data interpretation and critical revision. AA: study concept and design, methods development, clinical data interpretation and critical revision. RB: study concept and design and methods development. ZIW: study concept and design, data collection and analysis, clinical data interpretation, manuscript drafting, critical revision, and study supervision. All authors contributed to the article and approved the submitted version.

## Conflict of Interest

The authors declare that the research was conducted in the absence of any commercial or financial relationships that could be construed as a potential conflict of interest.

## Publisher's Note

All claims expressed in this article are solely those of the authors and do not necessarily represent those of their affiliated organizations, or those of the publisher, the editors and the reviewers. Any product that may be evaluated in this article, or claim that may be made by its manufacturer, is not guaranteed or endorsed by the publisher.
